# Allergie respiratoire et “sunko”: une association rare, à propos d'une observation

**DOI:** 10.11604/pamj.2014.18.271.4789

**Published:** 2014-08-04

**Authors:** Léon Kabamba Ngombe, Ignace Bwana Kangulu, Michel Kabamba Nzaji

**Affiliations:** 1 Unité de Toxicologie, Département de Santé Publique, Faculté de Médecine, Université de Kamina, Kamina, République Démocratique du Congo; 2 Département de Gynéco-Obstétrique, Faculté de Médecine, Université de Kamina, Kamina, République Démocratique du Congo

**Keywords:** Allergie respiratoire, Tabac sans fumé, Sunko

## Abstract

Il est rapporté dans ce texte un cas d'allergie respiratoire ayant fait probablement suite à une consommation d'un tabac sans fumé couramment utilisé en République Démocratique du Congo, le « Sunko ». Une rhinite et un asthme allergiques sont les cas. Cette observation permet d'attirer l'attention du monde scientifique à mettre sur pieds des études concernant la composition et les effets du Sunko pour appréhender cette association, puis d'informer l'opinion sur les dangers que courent les consommateurs de ce type de tabac et de faire la revue de la littérature.

## Introduction

Le tabagisme est la première cause de mortalité évitable dans le monde [1], ce qui en fait un enjeu majeur de santé publique. Quant à l'allergie respiratoire peut être une conséquence directe du tabac comme allergène du tractus respiratoire. De ce fait, elle est composée principalement de deux grandes pathologies à savoir les rhinites allergiques et l'asthme. Le tabac sans fumée(TSF) n'est pas sans danger [2]. En 2001, dans une étude menée en Afrique du Sud parmi les élèves noirs des écoles secondaires, la prévalence du tabac à priser était de 8,4% chez les filles, et de 3,9% chez les garçons [3]. Ainsi le « Sunko » est un TSF populaire après la cigarette, spécifique à la RD Congo qui a été utilisé depuis les ancêtres dès leurs bas âges jusqu’à nos jours dans certains milieux. Cependant, les risques potentiels de l'association entre l'allergie respiratoire et l'usage du Sunko demeurent inconnus dans la population. La RD Congo ne dispose pas des données épidémiologiques relatives à la consommation de ce dernier, le Sunko. Le but de ce travail est d'attirer l'attention de l'opinion scientifique à mettre en marche des études poussées afin de comprendre l'association rare entre l'allergie respiratoire et le tabac « Sunko », et sensibiliser l'opinion publique concernant le danger de l'usage de ce dernier.

## Patient et observation

Nous avons consulté une patiente âgée de 67 ans, qui a présenté comme plaintes dyspnée, toux, asthénie, sensation de nez bouché, fièvre. Le débit expiratoire de pointe pris en urgence a été de 200 ml. Dans les antécédents toxico-allergiques, elle est fumeuse du Sunko par la bouche et par le nez depuis l’âge de 13 ans. A 38 ans, elle a commencé à avoir des rhinorrhées, des crises des dyspnées pour lesquelles elle recevait du Prednisolone et du ventoline spray. 25 ans après, suite à des crises dyspnéiques récidivantes, une rhinite et un asthme allergique au TSF ont été suspectés.

**Figure 1:**
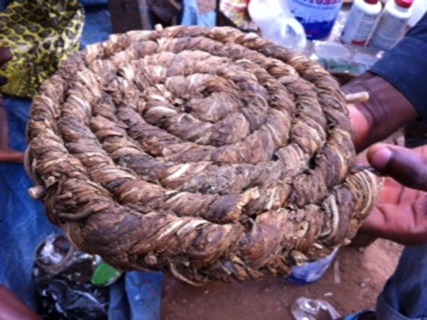
Un rouleau du tabac “Sunko” séché pour la vente

**Figure 2:**
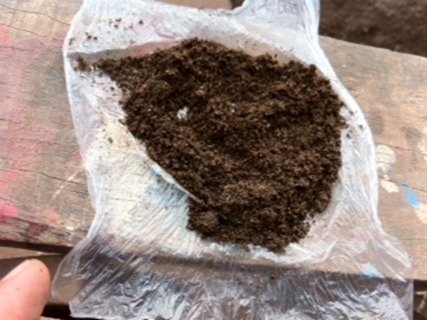
La forme pulvérisée du tabac “Sunko”, prête à l'utilisation (dans les narines ou en sublinguale)

A notre examen physique, la bouche était propre sans lésions buccales et gingivales observées; présence d'hypertrophie des cornets inferieurs dans les narines. Le thorax était dyspnéique, présence d'un tirage basithoracique, fréquence respiratoire était supérieur à 35 cycles /minutes, présence des râles sibilants dans les deux champs pulmonaires. Nous avons pensé à une crise d'asthme probablement allergique due au « Sunko » (Figure 1, Figure 2). Les radiographies des sinus et du thorax face ont été demandés, et les résultats étaient moins contributifs. La spirométrie avait confirmé le diagnostic de l'asthme et le questionnaire de SFR (Score for allergic rhinitis) celui de la rhinite allergique.

La patiente avait reçu des corticoïdes et du ventolin en spray. Une antibiothérapie de couverture (clamoxyl) avait également été instaurée. Des conseils de sensibilisation concernant l'arrêt du tabac sans fumée Sunko et ses dangers possibles ont été faits.

## Discussion

Selon l'OMS, le tabac est mortel sous toutes ses formes. Les études épidémiologiques mènent à la conclusion que la consommation de tabac est la première cause de décès évitable dans le monde [1]. Les allergies respiratoires(Rhinite, Asthme) sont dues à une exposition, même à faible niveau, à un agent sensibilisant, pouvant être chimique ou biologiques, d'origine animale ou végétale(INRS). Cependant dans notre milieu, les données concernant l'association rare entre le tabac « Sunko » et l'allergie respiratoire sont méconnus du public et des personnels soignants.

La patiente reçue a présentée des dyspnées, l'asthénie, la toux. L'examen physique a mis en évidence un tirage sus et basithoracique, présence des sibilants, des ronflants dans le thorax, ce qui signe une crise d'asthme confirmée en urgence par la mesure du débit expiratoire de pointe. Une semaine après, l'asthme a été confirmé par la spirometrie. Le test d'allergologie devrait être fait pour rechercher l'allergène. Cela n'a pas été le cas chez notre patiente.

Selon la littérature, le tabac sans fumée inclut le tabac à mâcher(ou à chiquer) et à priser. Le Sunko de la RDC fait partie de tabac sans fumée. Certains rapports affirment que l'usage de cette forme de tabac (TSF) augmente, particulièrement chez les adolescents et les enfants [4]. Ceci est vrai car notre patiente avait commencé à fumer le Sunko à l’âge de 13 ans par simple curiosité en imitant son grand père. En General, l'usage du tabac sans fumée est plus répandu chez les garçons que chez les filles mais il y a des endroits où les taux sont semblables [5]. Dans nos milieux, les études épidémiologiques ne sont pas disponibles.

Le tabac sans fumée n'est pas sans danger [2]. Ainsi, la teneur en nicotine est plus élevée que celle du tabac à fumer [6], et s'accompagne rapidement de la dépendance. En outre, la dépendance se traduit pour la plupart des consommateurs par des dizaines d'années d'exposition aux toxiques du tabac. Ceci pourrait expliquée la prise du Sunko sans arrêt de notre patiente durant plus de 40 ans.

Certains auteurs rapportent l'association entre le tabac sans fumée et le cancer de la bouche(ou de la cavité buccale) [4, 5]. Outre le cancer, on peut noter des lésions buccales, une altération de la santé dentaire et des gencives. Ceci n'a pas été le cas dans notre observation. La littérature ajoute encore qu'il est difficile de poser le diagnostic de la rhinite surtout professionnelle sur base de l'interrogatoire et de l'histoire clinique du patient [7, 8]. Par ailleurs, les symptômes de la rhinite allergique professionnelle sont les mêmes que les symptômes des rhinites révélant d'autres causes. D'où l'intérêt de l'utilisation du questionnaire SFAR (Score for allergic rhinitis) qui a une bonne sensibilité et spécificité [9], ce qui a été fait dans notre cas. Le délai de survenue de la rhinite allergique professionnelle est très variable de quelques semaines à plus de 10 ans après le début de l'exposition à l'agent causal [10]. Ceci est conforme à notre observation car la rhinite est apparue 20 ans après la prise du Sunko par le nez et la bouche.

Certaines études affirment que chez l'adulte, l'existence d'une rhinite augmente le risque de développer un asthme [11]. Notre observation vérifie cela. Quelques données publiées au sujet de la relation entre la rhinite et l'asthme dans la population générale révèlent une association fréquente de deux maladies, la rhinite précédant souvent l'apparition de l'asthme [12, 13]. Les asthmes à composante allergique apparaissent après un certain délai d'exposition (de plusieurs jours à plusieurs années) aux allergènes. Ils récidivent habituellement lors de chaque nouvelle exposition à l'agent causal, même à très faible concentration. Ceci est également valable pour notre cas, car toutes les fois que la patiente était en contact avec le Sunko, la rhinite et l'asthme réapparaissaient. Pour certains auteurs, la relation entre le tabagisme et le risque de sensibilisation reste controverse [8]. Néanmoins, notre observation montre que le tabac sans fumée peut être un allergène dangereux pour le système respiratoire. On a constaté également une hypo anosmie après plusieurs années d'utilisations du Sunko chez notre patiente. Ceci méritera d’être également étudié.

Plusieurs facteurs peuvent expliquer la popularité du tabac sans fumée chez les jeunes. Un de facteur est le fait que ce tabac est perçu comme étant moins nocif pour la santé que la cigarette et qu'il a été utilisé depuis longtemps par les ancêtres. Dans le cas qui nous concerne, c'est l'ignorance et la curiosité qui ont poussé l'adolescente à en faire usage. Cependant, nous n'avons pas su dire que le sunko est la véritable cause de l'allergie respiratoire compte tenu du manque des recherches approfondies sur ce tabac dans nos milieux. Néanmoins, cette association est rare et n'est pas documentée dans la littérature. Ceci attire notre attention et mérite d’être mentionnée, afin d'alerter l'opinion scientifique à mettre en marche des études approfondies concernant le Sunko et ses effets respiratoires afin d'appréhender cette association rare qui demeurent méconnus jusqu’à ce jour.

## Conclusion

Le tabac sans fumée est dangereux pour la santé respiratoire. Il s'avère important que des recherches soient mise en place pour appréhender cette association rare, afin de mieux prendre en charge nos patients et sensibiliser l'opinion public concernant le danger de l'usage de ce tabac dans nos milieux.
